# Tick saliva and its role in pathogen transmission

**DOI:** 10.1007/s00508-019-1500-y

**Published:** 2019-05-06

**Authors:** Patricia A. Nuttall

**Affiliations:** 1 Department of Zoology, University of Oxford, 11 Mansfield Road, OX1 3SZ Oxford, UK; 2 Centre for Ecology & Hydrology, Wallingford, Oxfordshire UK

**Keywords:** Ixodes ricinus, Vaccine, Saliva-assisted transmission, Tick-borne encephalitis virus, Borrelia burgdorferi

## Abstract

Tick saliva is a complex mixture of peptidic and non-peptidic molecules that aid engorgement. The composition of tick saliva changes as feeding progresses and the tick counters the dynamic host response. Ixodid ticks such as *Ixodes ricinus*, the most important tick species in Europe, transmit numerous pathogens that cause debilitating diseases, e.g. Lyme borreliosis and tick-borne encephalitis. Tick-borne pathogens are transmitted in tick saliva during blood feeding; however, saliva is not simply a medium enabling pathogen transfer. Instead, tick-borne pathogens exploit saliva-induced modulation of host responses to promote their transmission and infection, so-called saliva-assisted transmission (SAT). Characterization of the saliva factors that facilitate SAT is an active area of current research. Besides providing new insights into how tick-borne pathogens survive in nature, the research is opening new avenues for vaccine development.

## Introduction

Ticks are arthropods related to spiders and scorpions. Nearly 900 tick species are recognized of which 702 are ixodid ticks (family, Ixodidae) while 193 are argasid ticks (family, Argasidae) and a single species exists in the Nuttalliellidae [1]. All ticks go through a life cycle of egg, larva, nymph, and adult (female or male). Each postembryonic stage generally requires a blood meal before moulting to the next stage [2]. During blood-feeding, ticks acquire infections they may subsequently transmit when feeding again. When this occurs, ticks act as vectors of potential pathogens of humans and other animals [3]. In fact, ticks are believed to transmit the greatest variety of infectious agents of any blood-feeding vector. Notable diseases of humans caused by tick-borne pathogens include Lyme borreliosis, tick-borne encephalitis (TBE), tularemia, babesiosis, rickettsiosis, and human granulocytic anaplasmosis; however, tick-borne infectious agents (whether virus, bacterium, or protozoan) typically circulate in nature in a tick-vertebrate host-tick cycle without any apparent adverse effect on either the tick vector or the vertebrate host, be it reptile, bird, or mammal [4]. Hence, humans are usually oblivious to their presence making them hard to detect unless expensive monitoring procedures are implemented.

Tick-borne infectious agents are transmitted in the saliva of ticks. Importantly, the saliva of infected ticks is not simply a watery medium carrying pathogens but a complex mixture of hundreds of different molecules (Table 1). These saliva constituents help ticks obtain their blood meal while maintaining homeostasis. Increasing evidence reveals that tick-borne infectious agents exploit the activity of saliva molecules to promote their transmission, so-called saliva-assisted transmission (SAT). This means of facilitated transmission occurs both when infected ticks feed on a susceptible vertebrate host and when uninfected ticks feed on an infected vertebrate host. Ticks may even facilitate pathogen transmission via exosomes, extracellular vesicles in tick saliva [5]. In this review, the properties of tick saliva components and evidence of SAT are considered with particular focus on *Ixodes ricinus* (the wood or sheep tick), the most common tick species in northern and central Europe.

**Table 1:** Composition of tick saliva

Constituent	Examples
Water	Excess water from host bloodmeal
Ions	Na^+^, Cl^−^ (excess ions from host bloodmeal)
Non-peptidic molecules	Adenosine, prostaglandins, endocannabinoids, microRNAs
Tick peptides	Variegins, hyalomins, madanins
Tick proteins	Chitinases, mucins, ixostatins, cystatins, defensins, glycine-rich, hyaluronidases, Kunitz-type proteins, lipocalins, metalloproteases
Host proteins	Immunoglobulins, haptoglobin, transferrin
Exosomes	May contain microRNA, peptides, proteins

## Properties of tick saliva

Tick saliva is secreted from the relatively large and complex salivary glands of ticks during blood feeding. Besides water, saliva comprises a rich mixture of peptidic and non-peptidic molecules derived from the blood meal or synthesized by the salivary glands (Table 1). The volume and composition of saliva changes as feeding progresses, reflecting the uptake of blood (greatest during the final 24–48 h of engorgement) and dynamics of the host response. In large ixodid tick species, the total volume of saliva secreted during 10 days of engorgement may exceed 1 ml [6].

**Fig. 1:**
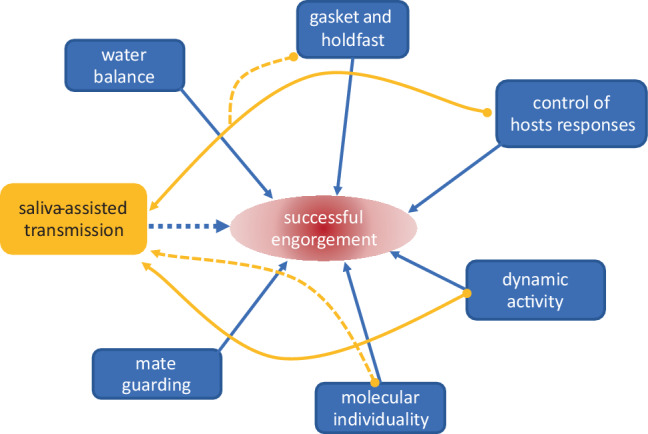
Major functions of tick saliva and their contribution to saliva-assisted transmission (SAT). Orange arrows indicate saliva functions contributing (solid line) or potentially contributing (dashed line) to SAT of tick-borne pathogens. Blue dashed line indicates potential for SAT to contribute to feeding success of infected ticks (modified from [7])

At least seven “functions” can be attributed to tick saliva: water balance, gasket and holdfast, control of host responses, dynamics, individuality, mate guarding, and SAT [7]. Given that ixodid ticks increase their body mass 10-fold to 200-fold during engorgement, excretion of water and ions extracted from the blood meal is essential to maintain tick homoeostasis [6]. Ensuring attachment is maintained during the long feeding period of ixodid ticks, and that nothing leaks out from the feeding site, is the role of the cement plug formed by polymerization of glycine-rich proteins secreted after initial attachment [8]. The attachment process severs tissues including nerves, causing pain and provoking host hemostatic (vasoconstriction, blood platelet aggregation, and fibrin clot formation), inflammatory, and immune responses [9, 10]. Most of the constituents of tick saliva function to counter these host responses. Such bioactive tick saliva molecules include analgesic bradykinin inhibitors, antihemostatic prostaglandins, vasoconstriction modulators, platelet activation and aggregation inhibitors, anticoagulants, anti-inflammatory proteins, immunomodulators and wound healing inhibitors [11–16]. As the host responses are dynamic (changing during the course of tick attachment, preparation of the feeding pool, and engorgement) so too is the saliva, changing in composition to meet the different needs of attachment, and slow and rapid feeding phases, and to counter the dynamic host responses [17–19]. Examination of single salivary glands of adult female *I. ricinus *confirmed expression of different clusters of genes at different times of feeding but also revealed differences between individual ticks [20]. As feeding ticks often cluster together on a host, they have the potential to pool their molecular individuality, which may help them feed [7]. Sharing of saliva resources has been demonstrated for male ticks as they “mate guard” their female mate [21]. Although mate guarding is not an option for species such as *I. ricinus* that mate off the host, sharing saliva resources is a possibility for all tick species in SAT of tick-borne pathogens (Fig. 1).

## Saliva-assisted transmission (SAT)

The ability of saliva to enhance the infectivity of an arthropod-borne pathogen was first described for the sandfly, *Lutzomyia longipalpis*, and the protozoan parasite, *Leishmania major* [22]. Follow-up of this discovery led to the finding that non-viremic transmission of a tick-borne virus between infected and uninfected ticks co-feeding on aviremic guinea pigs could be reproduced by inoculating the experimental guinea pigs with the virus mixed with an extract of uninfected tick salivary glands [23, 24]. The phenomenon was named saliva-activated transmission which was subsequently corrected to saliva-assisted transmission, defined as “the indirect promotion of arthropod-borne pathogen transmission via the actions of arthropod saliva molecules on the vertebrate host” [25]. There is now extensive evidence that saliva of blood-feeding arthropods (mosquitoes, sandflies, ticks) affects the transmission of vector-borne viruses, bacteria, and protozoa in addition to simply acting as a transfer medium [12, 15, 26–30]. Considerable evidence relates to the immunomodulatory activities of tick saliva and, more recently, the dynamics of tick saliva-induced control of host responses (Fig. 1); however, tick cement may contribute to SAT (*vide infra*) as may the individuality of ticks each with its unique sialome [7].

This review focuses on pathogens transmitted by *I. ricinus*, the vector of numerous human pathogens, most notably TBE virus and certain species of the *Borrelia burgdorferi *sensu lato (s. l.) complex that cause Lyme borreliosis [31, 32]. Evidence of SAT is also considered involving the sympatric and parapatric species, *I. persulcatus* (found in parts of northern Europe and northern to north-eastern Asia), and the allopatric species of North America, *I. scapularis* [33–37].

### Tick-borne encephalitis virus

At least 10 viruses (including subtypes and strains) transmitted by *I. ricinus* have been recorded in Europe [38]. The most important of these as a human pathogen is TBE virus, a species belonging to the virus family, Flaviviridae [39]. The TBE virus is distinguished into 3 subtypes: European, Siberian, and Far Eastern [40] and two additional subtypes, found in Siberia and the Himalayas, are proposed [41, 42]. In most of Europe, TBEV-Eu is the prevailing subtype, transmitted primarily by *I. ricinus*, and small rodents are the principal natural host [32]. Despite ample evidence that tick saliva facilitates TBE virus transmission and infection, the mechanism and active saliva ingredients have not been identified for TBE virus or any other tick-borne virus (Table 2).

**Table 2:** Saliva-assisted transmission of human pathogens transmitted by *Ixodes ricinus*

Pathogen	Saliva factor^a^	Activity	Reference
TBE virus (Far Eastern subtype)	*Dermacentor marginatus *adult SGE day 5	“Adjuvant” activity enhancing transmission to ticks	[43]
TBE virus (European subtype)	*I. ricinus* female SGE day 5	Enhanced transmission from guinea pigs to ticks	[44]
TBE virus (European subtype)	Co-feeding infected and uninfected *I. ricinus*	Non-viremic transmission	[45–47]
TBE virus (European subtype)	*I. ricinus *female saliva day 6	Enhanced replication in murine spleen dendritic cells ex vivo	[48]
Louping ill virus	Co-feeding infected and uninfected *I. ricinus*	Non-viremic transmission	[49]
*Borrelia burgdorferi *s.s.	Co-feeding infected and uninfected *I. ricinus*	Non-systemic transmission	[50]
*Borrelia burgdorferi *s.s.; *B. lusitaniae*	*I. ricinus *nymph SGE day 2–3	Enhanced spirochete load in mice	[51]
*Borrelia afzelii*	*I. ricinus *female SGE day 5	Enhanced infection of mice and feeding ticks	[52]
*Borrelia burgdorferi *s.s.	*I. ricinus *female saliva/SGE day 6	Enhanced transmission from mice to ticks	[53]
*Borrelia burgdorferi *s.s.	Salp15 Iscap	Facilitates transmission from ticks to mice^b^	[54]
*Borrelia burgdorferi *s.s.	Salp15 Iric-1	Facilitates needle infection of immune mice	[55]
*Borrelia burgdorferi *s.s.; *Borrelia garnii*	Salp15 Iscap and Iric-1	In vitro protection against complement-mediated killing	[56]
*Borrelia burgdorferi* s.s.	Salp15 Iric-1	In vitro suppression of borrelia-induced keratinocyte inflammation	[57]
*Borrelia burgdorferi *s.s.	TSLPI^c^	Facilitates transmission from ticks to mice and from mice to ticks^d^	[58]
*Borrelia burgdorferi *s.s.; *B. garinii*	TSLPI Iric	In vitro reduction of complement-mediated killing	[59]
*Borrelia garnii*	Salp20	In vitro protection against complement-mediated killing	[60]
*Borrelia burgdorferi *s.s.	tHRF^e^	Promotes late stage feeding and thereby facilitates tick to host transmission	[61]
*Borrelia burgdorferi *s.s.	Sialostatin L2^e^	Increases level of skin infection following syringe inoculation	[62]
*Borrelia burgdorferi *s.s.	Salp25D^e^	Facilitates transmission from mice to ticks	[63]
*Borrelia burgdorferi *s.s.	BIP^f^	In vitro inhibition of OspA and OspC-induced B cell proliferation	[64]
*Anaplasma phagocytophilum*	Sialostatin L2^e^	Promotes infection of mice by impairing inflammasome formation	[65]
*Francisella tularensis*	*I. ricinus *female SGE day 5	Accelerated proliferation in mice	[66]

*tHRF* tick histamine release factor

^a^Salivary gland extract (SGE) or saliva collected at specified day of feeding, or specific saliva protein; recombinant proteins Iscap from *I. scapularis *and Iric from *I. ricinus*. Most studies involving specific saliva proteins are with *I. scapularis*-derived recombinant proteins.

^b^Direct evidence of Salp15-assisted transmission of *B. burgdorferi *has only been reported for *I. scapularis *[54]

^c^Tick salivary lectin pathway inhibitor

^d^Direct evidence of TSLPI-assisted transmission of *B. burgdorferi *has only be reported for *I. scapularis *[58]

^e^Derived from *I. scapularis*

^f^B cell inhibitory protein from *I. ricinus*

Indirect evidence of SAT of TBE virus was first provided by experimental studies designed to test whether non-viremic transmission of Thogoto virus could be reproduced with TBE virus [23]. Uninfected *I. ricinus* nymphs became infected with TBE virus when co-feeding (in separate retaining chambers) with infected* I. ricinus *adult ticks on guinea pigs that did not develop a patent viremia [45]. Similar observations were reported when infected and uninfected ticks fed together; at the time, this was referred to as transptialonic transmission [67]. Of major significance was the demonstration of non-viremic transmission with natural host species of TBE virus and *I. ricinus* (Table 2). Wild-caught field mice (*Apodemus flavicollis* and *A. agrarius*), bank voles (*Myodes glareolus*), pine voles (*Pitymys subterraneus*), hedgehogs (*Erinaceaus europaeus*) and pheasants (*Phasianus colchicus*), all of which had no evidence of prior exposure to TBE virus (absence of specific neutralizing antibodies) were infested with TBE virus-infected *I. ricinus* adult females and uninfected *I. ricinus* nymphs (in separate chambers). Although field mice showed undetectable or comparatively low levels of virus infection, they produced the greatest yield of infected ticks. By contrast, pine voles produced high levels of viremia and relatively few infected ticks because the animals died [46]. The study provided the first evidence that TBE virus transmission from infected to uninfected ticks occurs during co-feeding on natural host species and is independent of the development of viremia. Indeed, models of TBE virus survival based on estimates of the basic reproduction number (R_0_) indicated that TBE virus cannot survive in nature without non-viremic/co-feeding transmission [68].

Co-feeding transmission takes advantage of the fact that ticks show a typical negative binomial distribution on their hosts: at any one time, a small number of individual animals are heavily infested with ticks whereas the majority of the host population are uninfested or support low numbers of ticks [69]. Moreover, ticks are gregarious feeders; for example, >90% immature *I. ricinus *feed together on the ears of rodents or around the bill of birds [70]. Minimization of the distance between co-feeding infected and uninfected ticks, as a result of feeding aggregation, facilitates non-viremic transmission [67, 71, 72]. Co-feeding/non-viremic transmission may also aid survival of TBE virus in natural foci of infection by amplifying low levels of transovarial transmission of the virus from one tick generation to the next [47]. Survival is also aided by the ability of hosts immune to TBE virus to support co-feeding/non-viremic transmission, although at reduced levels compared with transmission involving non-immune natural host species [71]. Given that non-viremic transmission is facilitated by SAT, the natural history of TBE virus demonstrates the crucial role tick saliva molecules play in maintaining a major human pathogen.

Evidence that TBE virus transmission is enhanced by factors associated with the salivary glands of uninfected ticks was first indicated experimentally when tick-infested guinea pigs were inoculated with a mixture of TBE virus and salivary gland extract (SGE) derived from uninfected partially fed adult *Dermacentor marginatus *(Table 2). The SGE was considered to act as an adjuvant, increasing the number of *Dermacentor *spp. nymphs that became infected while feeding on the non-viremic guinea pigs [43, 73]. When guinea pigs infested with uninfected *Rhipicephalus appendiculatus *nymphs were inoculated with a mixture of TBE virus and SGE, there was a 3-fold to 5‑fold increase in the number of infected ticks from animals inoculated with TBE virus and SGE from partially fed *I. ricinus *ticks compared with virus alone or virus plus SGE from unfed ticks [44]. This was the first clear evidence that TBE virus transmission is enhanced by factors associated with the salivary glands of feeding ticks, and that these factors may explain the efficient transmission of TBE virus between infected and uninfected co-feeding ticks in natural non-viremic transmission.

Attempts to identify the salivary gland factor(s) assisting TBE virus or any other tick-borne virus have so far been unsuccessful. A study with Thogoto virus demonstrated similar SAT dynamics for saliva and SGE collected from the same individual uninfected ticks at different days of feeding [74]. Maximum activity was observed with saliva at day 6 of feeding and days 6–8 for SGE. The similar dynamics of SAT activity are a strong indication that the SAT factor(s) is synthesized in the salivary glands during feeding and secreted into the skin feeding site in tick saliva. Interestingly, SAT of tick-borne viruses has only been demonstrated with arthropod species that are competent vectors. Thus, SGE of *I. ricinus* does not promote SAT of Thogoto virus (for which *I. ricinus* is not a competent vector) although SAT occurs with TBE virus [44, 75, 76]. This implies that the mechanism underlying SAT differs for different vector-virus associations.

Further studies using Thogoto virus provided additional insights into the mechanism of SAT of tick-borne viruses. To examine whether the SAT factor has a direct or an indirect effect on Thogoto virus, the time interval was varied between virus inoculation and injection of SGE into the same skin site of experimental guinea pigs. Similar levels of SAT were observed when virus + SGE were inoculated together compared with an interval between SGE injection followed by virus inoculation of 72 h or less; when the interval was 96 h the level of SAT was halved. In the converse experiment, SAT levels were similar when the interval between virus inoculation followed by SGE injection was 0 h, 24 h, or 48 h but was significantly reduced at higher intervals [77]. Moreover, SAT is a localized effect rather than a generalized response in the host: when virus + SGE were inoculated into different skin sites of experimental guinea pigs, SAT was not observed. In addition, in vitro and in vivo titrations of Thogoto virus mixed with SGE showed no effect on infectivity [77]. These observations indicate that SAT (at least for Thogoto virus) results from the effect of saliva in the feeding site rather than from a direct effect on the virus and the beneficial effect, whatever it may be, lasts for several days. They also suggest some dynamic variance: virus and SAT factor do not need to be delivered to the feeding site simultaneously to be effective. This window of activity may be significant as tick-borne virus transmission occurs within 24 h of tick attachment, and possibly within the first hour of feeding [78–80]. The onset of feeding enhances virus replication in the salivary glands [67, 81, 82]. Once initiated, tick-borne virus transmission may continue throughout the feeding period [78]. Hence, tick-borne viruses may experience a broad spectrum of saliva-mediated activities in the skin site of tick feeding although SAT activity has only been reported with saliva/SGE collected at least 4 days after commencement of feeding (Table 2). In addition, the cement plug may act as a bolus of infection for both TBE virus and *B. burgdorferi* s. l. Removal of attached infected ticks while leaving the cement cone embedded in the skin resulted in infection with TBE virus within 1 h of attachment and after 20–22 h of attachment in the case of *B. burgdorferi *s. l. and *I. persulcatus *[83].

The SAT factor(s) promoting Thogoto virus transmission by its principal vector, *R. appendiculatus, *appears to be proteinaceous as activity was lost following protease treatment [84]. It remains to be determined whether these features apply to SAT of other tick-borne viruses including TBE virus although it seems likely. Enhanced replication of TBE virus occurs in murine bone marrow dendritic cells treated with the feeding-induced saliva protein, sialostatin L2 from *I. scapularis* nymphs [85]; however, sialostatin L2 has not been characterized in the natural vector of TBE virus, *I. ricinus*. Enhanced virus replication and survival in dendritic cells treated with saliva of partially fed adult female *I. ricinus *is attributed to saliva-induced modulation of the pro-survival phosphatidylinositol 3 kinase (P13)/Akt signal transduction pathway by an as yet unknown mechanism [48, 86].

In contrast to tick-borne viruses, at least two SAT factors have been identified for mosquito-borne viruses. A saliva protein from *Aedes aegypti*, CLIPA3 protease, promotes replication and dissemination of dengue type 2 viruses in interferon knockout mice through cleavage of extracellular matrix proteins; resulting dermal liquefaction is thought to facilitate virus infection of skin immune cells [87]. In a similar mouse-mosquito model, a 15 kD *A. aegypti* saliva protein, LTRIN, augmented pathogenesis of Zika virus by interfering with signalling through the lymphotoxin-β receptor [88]. In cultures of human keratinocytes, a 34 kD *A. aegypti *saliva protein of unknown function enhanced dengue type 2 virus replication [89]. Further studies are needed to determine whether this saliva protein promotes initial skin infection following mosquito-borne transmission of dengue virus. Progress in identifying SAT factors of mosquito-borne viruses will hopefully inspire research on the saliva molecules promoting transmission of TBE virus and other tick-borne viruses.

### *Borrelia burgdorferi* sensu lato

The bacterial complex, *Borrelia burgdorferi* s. l., includes 18 species of which 3 species commonly infect humans causing Lyme borreliosis: *B. burgdorferi *sensu stricto (s.s.), *B. afzelii*, and *B. garinii* [90]. As in the case of tick-borne viruses (*vide supra*), the first hint that saliva plays an enhancing role in the transmission of *B. burgdorferi *s. l. was in studies showing efficient non-systemic transmission of *B. burgdorferi *s.s. between infected nymphs and uninfected *I. ricinus *larvae co-feeding on uninfected laboratory mice [50]. In subsequent studies, experimental mice were inoculated with a mixture of either *B. burgdorferi* s.s. (isolated from *I. scapularis*) or *B. lusitaniae *(isolated from *I. ricinus*) and SGE prepared from either uninfected *I. scapularis *or *I. ricinus *partially fed nymphs (Table 2). The SGE promoted infection of the mice; the effect was tick-spirochete species-specific [51]. Similar results were observed using *B. afzelii *isolated from *I. ricinus *and SGE from partially fed adult female *I. ricinus* [52]. Inoculation of mice with saliva mixed with *B. burgdorferi* s.s. (isolated from *I. ricinus*) showed the same enhancing effect as with SGE [53].

Studies on *B. burgdorferi *s. l. were the first to identify a tick-derived SAT factor, Salp15. This saliva protein was originally identified as a glycosylated 15 kD feeding-inducible protein (at a concentration of 1 µg/ml saliva) that inhibits CD4^+^ T cell activation, the first *I. scapularis* protein associated with the immunosuppressive activity of tick saliva [91]. The Salp15 binds to the CD4 coreceptor of mammalian T cells, inhibiting subsequent receptor ligand-induced early cell signalling, which explains its immunosuppressive activity and specificity for CD4 T cells [92]; however, Salp15 also binds to the C‑type lectin receptor, dendritic cell-specific intercellular adhesion molecule-3-grabbing non-integrin (DC-SIGN, also known as CD209), inhibiting Toll-like receptor (TLR)-induced production of proinflammatory cytokines (including cytokines induced by *B. burgdorferi*) and dendritic cell-induced T cell activation [93]. The activity of Salp15 appears long lasting, possibly due to Salp15-induced upregulation of CD73 (5′-ectonucleotidase) in regulatory T cells, which increases production of adenosine, a recognized immunosuppressant [94].

An initial indication that Salp15 might be involved in SAT was the selective enhancement of *salp15 *expression observed in *I. scapularis* nymphs infected with *B. burgdorferi* s.s. [54]. Further studies showed that Salp15 protected *B. burgdorferi* s.s. from antibody-mediated killing by binding to the outer surface protein, OspC, of the spirochete. This lipoprotein is expressed when the spirochete infects the tick salivary glands and during the early stages of vertebrate host infection [95]. Knockdown of *salp15* in infected *I. scapularis *markedly reduced the spirochete load in mice on which the nymphs fed (levels of infection were similar in the salivary glands of engorged *salp15*-repressed and control nymphs). These observations were replicated using white-footed mice, *Peromyscus leucopus* (natural hosts of *I. scapularis *and *B. burgdorferi* s.s. in North America), even when the mice were immune to the spirochete. The gene knockdown experiments provide the first direct evidence that Salp15 promotes tick-borne transmission of *B. burgdorferi* [54]. Protection of Salp15-immunized mice against *I. scapularis*-transmitted* B. burgdorferi* s.s. provides further supportive data [96].

Following the discovery of Salp15-assisted transmission of *B. burgdorferi* s.s. by *I. scapularis*, other *Ixodes *species were investigated. Orthologues of Salp15 were found in all *B. burgdorferi* s. l. vector species including 3 homologues in *I. ricinus *[97]. One of these homologues (Salp15 Iric-1) shares 82% homology with the *I. scapularis *protein and is highly expressed at 3 days of feeding. Like Salp15 from *I. scapularis, *Salp15 Iric-1 binds to OspC. Mice antibody-positive for *B. burgdorferi *s.s. (strain N40) were more susceptible to infection with strain N40 preincubated with Salp15 Iric-1 compared with needle challenge with the untreated strain; however, Salp15 Iric-1 did not facilitate infection of *B. afzelii*-immune mice with *B. afzelii *[55].

Comparison of recombinant Salp15 derived from *I. ricinus *and *I. scapularis *revealed protection of serum-sensitive *B. garinii *and* B. burgdorferi* s.s. strains of intermediate sensitivity against complement-mediated killing by normal human serum. The effect was significantly stronger for Salp15 from *I. ricinus* compared with *I. scapularis *[56]. Interestingly, Salp15 Iric-1 binding to *B. burgdorferi *s.s. OspC conferred protection against antibody-mediated killing whereas binding of Salp15 Iric-1 to OspC from *B. garinii *and *B. afzelii *was not protective even though binding affinities were similar [55]. The *I. ricinus* Salp15 also shows an antialarmin effect on human primary keratinocytes in vitro, suppressing inflammation induced by *B. burgdorferi *s.s. infection [57].

The differential effects of Salp15 in relation to complement and antibody-mediated killing may help explain spirochete-vector species specificity [51]. If these effects of Salp15 (and possibly other saliva proteins) occur when ticks feed on a natural host or a human, the ensuing inhibition of cutaneous innate immunity (including suppression of immune cell recruitment) will most likely promote *B. burgdorferi *transmission and infection of the host. Note the effects will also help the infected tick by suppressing the undesirable (from the tick’s point of view) host responses to infection by the pathogen.

Characterization of Salp15-assisted transmission of *B. burgdorferi *demonstrates the importance of classical (antibody-dependent) and alternative (antibody-independent) complement pathways in vertebrate host control of the spirochete; however, *Ixodes* vector species also interfere with the lectin (antibody-independent) complement cascade [58, 59]. The lectin pathway is activated when the pattern recognition molecules of the lectin pathway, ficolins and collectins (including mannan-binding lectin), bind to highly glycosylated pathogen-associated molecular patterns on the surface of pathogens [98]. Tick salivary lectin pathway inhibitor (TSLPI) is a feeding-induced 8 kD saliva protein first identified in *I. scapularis* and initially designated P8 [99]. Unlike Salp15, TSPLI does not bind to *B. burgdorferi *but instead interacts with the lectin pathway pattern recognition molecules, inhibiting complement activation and reducing complement-mediated lysis of *B. burgdorferi *s. l*. *[58]. In vitro, TSLPI impairs complement-mediated chemotaxis and phagocytosis of *B. burgdorferi *s. l*. *by neutrophils. Heat-inactivated TSLPI antiserum substantially reduces the complement inhibitory activity of *I. scapularis* SGE in vitro, indicating that TSLPI is a dominant complement inhibitor in tick saliva. Heat-inactivated TSLPI antiserum also reduces the lectin pathway inhibitor activity of *I. scapularis* SGE, showing that native TSLPI is a major inhibitor of tick-borne *B. burgdorferi*-mediated complement activation through the lectin pathway. Compared with uninfected ticks, infection of *I. scapularis *nymphs with *B. burgdorferi* s.s. results in significantly higher *TSLPI* mRNA levels in salivary glands 24 h after tick attachment [58]. The timing neatly coincides with tick-borne delivery of *Borrelia* into the feeding site. Knockdown of *TSLPI* in nymphs reduced the *Borrelia *load in infected ticks fed on uninfected mice for 72 h. Transmission of *B. burgdorferi* s.s. to uninfected mice by *TSLPI *knockdown nymphs resulted in a significantly lower spirochete load in skin 7 days postinfestation and reduced dissemination at 21 days. Similar effects were observed when infected nymphs were fed on mice which had been passively administered TSLPI antiserum [58]. Acquisition of *B. burgdorferi* s.s. by *I. scapularis *larvae was impaired on infected TSLPI-immune mice and the *Borrelia *load was reduced in nymphs moulted from the larvae compared with the controls. Results of the *TSLPI *knockdown experiments provide direct evidence of TSLI-assisted transmission of *B. burgdorferi* s.s. by *I. scapularis *supported by abrogation of the effect in TSLPI-immune mice. As killing of *Borrelia *by the lectin complement pathway occurs in the absence of *B. burgdorferi* antibodies, the protective effect of TSLPI is most likely a critical factor in tick-borne *B. burgdorferi *infection of naïve humans.

Bioinformatic analysis of *Ixodes *species indicates TSLPI comprises a family of saliva proteins [59]. An orthologue from *I. ricinus*, upregulated during feeding, inhibits the lectin complement pathway and protects *B. burgdorferi* s.s. and *B. garinii *from complement-mediated killing in vitro. Studies of the role of *I. ricinus* TSLPI in tick-borne transmission of* B. burgdorferi *have not been reported to date. Additionally, numerous inhibitors of the alternative complement pathway have been recognized in *Ixodes *ticks (including *I. ricinus*), the first of which was named *Ixodes scapularis *anti-complement (ISAC). The ISAC-related proteins (designated the IxAC family) target properdin, a positive activator of the alternative complement pathway [100]. The family includes Salp20 (*I. scapularis *salivary protein 20) which provides partial protection of complement sensitive *B. garinii* against lysis by normal human serum in vitro [60]. Although the IxAC family may aid complement-sensitive *B. burgdorferi* strains during the early stages of skin invasion in mammals, their role in tick-borne borrelia transmission has not been explored.

Histamine is an important tick deterrent [101]. Not surprisingly, ticks have evolved saliva molecules that control host-derived histamine [102]; however, what is surprising is that certain tick species deliberately release histamine into the tick feeding site [103]. The tick histamine release factor (tHRF) of *I. scapularis* appears to aid the late (rapid) phase of feeding and also to promote *B. burgdorferi *s.s. transmission [61]. Expression of this feeding-induced 24 kD protein is enhanced in *B. burgdorferi *infected *I. scapularis *nymphs, knockdown of *tHRF-*impaired feeding and borrelia transmission. As tHRF is critical for *I. scapularis* feeding irrespective of *B. burgdorferi* infection, and preferentially expressed 48–72 h post-tick attachment (while *B. burgdorferi *transmission begins 36–48 h post-tick attachment), it seems likely that the effect on borrelia transmission is due to the ability of tHRF to promote engorgement rather than to a specific interaction as with Salp15 (*vide supra*). Indeed, it is thought that tHRF promotes engorgement by enhancing blood flow into the tick feeding site during the final 24 h of feeding when ~50% of the blood meal is taken up. Conceivably, the vasodilatory effect of histamine may aid dissemination of borrelia injected by the tick into the feeding site [61]. As yet, an *I. ricinus* orthologue of *I. scapularis *tHRF has not been characterized.

Evidence that the cysteine protease, sialostatin L2, is a SAT factor was first observed following syringe inoculation of *I. scapularis *sialostatins and *B. burgdorferi* s.s. into the skin of mice. In the presence of sialostatin L2, the number of spirochetes in the skin increased almost 6‑fold, 4 days post-inoculation, whereas sialostatin L had no effect [62]. Sialostatin L2 does not bind directly to spirochetes and appears not to affect *B. burgdorferi* s.s. growth in culture [62]. In borrelia-infected murine dendritic cells, sialostatin L2 inhibited borrelia-induced chemokine production and Toll-like receptor signalling pathways [85, 104]. Thus, sialostatin L2 may assist *B. burgdorferi *by suppressing the pathogen-induced inflammatory response. An orthologue of sialostatin L2 has not been reported for *I. ricinus *although a putative orthologue has been recorded in *I. persulcatus *[105].

Most SAT factors are considered in relation to pathogen transmission from infected ticks to a susceptible vertebrate host [106]; however, some saliva proteins appear to aid in the acquisition of the pathogen by uninfected feeding ticks. One such protein is Salp25D, an antioxidant protein identified in both the salivary glands and midgut of *I. scapularis* [63]. Knockdown of salivary gland *Salp25D *dramatically reduces acquisition of *B. burgdorferi* s.s. by feeding *I. scapularis *nymphs and larvae but does not affect spirochete transmission from infected nymphs to uninfected mice. Ticks failed to acquire *B. burgdorferi *when fed on mice immunized against Salp25D. In vitro, oxygen radicals produced by activated neutrophils reduce the viability of *B. burgdorferi*, whereas in the presence of recombinant Salp25D or adult *I. scapularis *saliva, viability was unaffected. Thus, it appears that Salp25D assists in transmission of *B. burgdorferi *from infected mice to uninfected ticks by protecting borrelia from the toxic products of neutrophils activated by tick feeding.

An 18 kD B cell inhibitory protein (BIP) identified in the salivary glands of *I. ricinus*, inhibits B lymphocyte proliferation induced by *B. burgdorferi *outer surface proteins, OspA and OspC [64]. As these lipoproteins play essential roles in *B. burgdorferi *infection of the tick midgut and in tick-borne transmission to a vertebrate host, respectively, BIP may act as a localized SAT transmission factor facilitating both tick-borne transmission and tick acquisition of the spirochete.

A number of other saliva proteins, mainly from *I. scapularis*, affect *B. burgdorferi* transmission. These include subolesin [107, 108] and calreticulin [109]. Their effect appears to result from their critical role in tick physiological processes rather than acting as SAT factors per se. Histone H4 isolated from *I. ricinus* SGE has a dissociating effect on human primary fibroblast and antimicrobial properties although it does not affect *B. burgdorferi *s.s. [110]. These characteristics suggest that histone H4 plays a role in formation of the tick feeding pool within the skin and thereby may aid borrelia transmission.

### Other human pathogens transmitted by *Ixodes ricinus*

Louping ill virus, a close relative of TBE virus, causes disease in sheep and red grouse (*Lagopus scotica*) in the UK, and is an occupational health risk for veterinarians, game keepers, and farm workers [111]. The discovery that mountain hares (*Lepus timidus*) support non-viremic transmission of louping ill virus (even when the hares have antibodies to the virus), identified the reservoir host of this virus [49]. This has led to large-scale culling of mountain hares on Scottish moorland managed for red grouse, which has given rise to considerable controversy [112].

The intracellular bacterium, *Anaplasma phagocytophilum*, has long been known as the etiological agent of tick-borne fever affecting ruminants and was only recognized relatively recently as the pathogen causing human granulocytic ehrlichiosis [113]. Although studies have shown that sialostatin L2 from *I. scapularis *plays a role in transmission of *A. phagocytophilum*, the role of *I. ricinus *saliva proteins in *A. phagocytophilum *transmission, including orthologues of sialostatin L2, has not been reported (Table 2).

*Bartonella *spp. are linked with an increasing number of human diseases of which the most common are the multiple clinical symptoms associated with cat scratch disease, ocular infections, and endocarditis caused by *B. henselae*. Cat fleas are the principal vector of *B. henselae*; however, *I. ricinus *is also a vector of this intracellular bacterium although the epidemiological significance of tick-borne transmission is unknown [114]. A serine protease inhibitor from *I. ricinus* (IrSPI) affects *B. henselae *salivary gland infection and tick feeding success [30]. The role of IrSPI in transmission of *B. henselae *has not been determined.

The etiological agent of tularemia, *Francisella tularensis*, is an intracellular bacterium endemic in European rodent populations and hares (*Lepus europaeus*), and transmitted directly by contact and inhalation, and by ticks [115]. Mice injected with a mixture of the live vaccine strain of *F. tularensis* and SGE supported accelerated proliferation of the bacterium in skin and other target organs (Table 2). As Th1-dependent cell-mediated immunity is critical for protection against infection with *F. tularensis*, the observed SGE-induced polarization to a Th2 cytokine profile most likely benefited the tularemic bacteria [66].

## Future developments

Just as sandfly saliva-leishmania was the forerunner to discoveries of the role of tick saliva in tick-borne pathogen transmission (*vide supra*), so vaccine development using sandfly saliva proteins to control leishmaniasis is pioneering a new approach to controlling arthropod-borne pathogens [116, 117]. For mosquito-borne pathogens, this has developed as far as a phase 1 clinical trial in humans of a vaccine comprising 4 mosquito-derived saliva antigens [118]. Protection against lethal tick-borne challenge with TBE virus by immunization of mice with a tick cement protein, demonstrates the potential of this approach for controlling tick-borne pathogens [119]. Tick saliva proteins that facilitate pathogen transmission have been identified as candidates for development of anti-tick vaccines [30, 120]. An alternative strategy is to target tick proteins that have a significant physiological role. For example, salivary gland aquaporins provide water transmembrane channels crucial for water homeostasis during blood feeding. Survival of *I. ricinus *larvae fed on rabbits immunized with a vaccine comprising recombinant *I. ricinus *aquaporin was significantly reduced [121]. For leishmania vaccine development, the most promising results involve a dual pathogen plus vector vaccine design [117]. Clearly there is much to do in the development of new, effective and efficient vaccines for controlling ticks and important major tick-borne infections. At least it is now realized that “saliva is mightier than the needle”—future vaccine development needs to include challenge with infected ticks and not the typical needle and syringe inoculation of the challenge pathogen [122–124].
